# Evolution of RNA Viruses: Reasons for the Existence of Separate Plus, Minus, and Double-Strand Replication Strategies

**DOI:** 10.3390/v16071081

**Published:** 2024-07-05

**Authors:** Hyunjin Park, Paul G. Higgs

**Affiliations:** Department of Physics and Astronomy, McMaster University, Hamilton, ON L8S 4L8, Canada; parkh54@mcmaster.ca

**Keywords:** plus-strand viruses, minus-strand viruses, double-strand viruses, viral evolution, multiplicity of infection, evolutionary theory

## Abstract

Plus, minus, and double-strand RNA viruses are all found in nature. We use computational models to study the relative success of these strategies. We consider translation, replication, and virion assembly inside one cell, and transmission of virions between cells. For viruses which do not incorporate a polymerase in the capsid, transmission of only plus strands is the default strategy because virions containing minus strands are not infectious. Packaging only plus strands has a significant advantage if the number of RNA strands produced per cell is larger than the number of capsids. In this case, by not packaging minus strands, the virus produces more plus-strand virions. Therefore, plus-strand viruses are selected at low multiplicity of infection. However, at high multiplicity of infection, it is preferable to package both strands because the additional minus virions produced are helpful when there are multiple infections per cell. The fact that plus-strand viruses are widespread while viruses that package both strands are not seen in nature suggests that RNA strands are indeed produced in excess over capsids, and that the multiplicity of infection is not sufficiently high to favor the production of both kinds of virions. For double-strand viruses, we show that it is advantageous to produce only plus strands from the double strand within the cell, as is observed in real viruses. The reason for the success of minus-strand viruses is more puzzling initially. For viruses that incorporate a polymerase in the virion, minus virions are infectious. However, this is not sufficient to explain the success of minus-strand viruses, because in this case, viruses that package both strands outcompete those that package only minus or only plus. Real minus-strand viruses make use of replicable strands that are coated by a nucleoprotein, and separate translatable plus strands that are uncoated. Here we show that when there are distinct replicable and translatable strands, minus-strand viruses are selected.

## 1. Introduction

Many types of RNA virus exist that package and transmit their genetic sequences in different ways. Viruses are often classified according to the Baltimore classification [1]. Here we focus on plus-strand RNA viruses, minus-strand RNA viruses, and double-strand RNA viruses, and we do not consider retroviruses and DNA viruses. All viruses require plus and minus strands in one form or another. A plus strand has the same sense as an mRNA and can be translated to produce viral proteins. A minus strand is complementary to a plus strand and cannot be translated, but is necessary as a template for plus-strand synthesis. A plus-strand virus is one in which the plus strand is packaged into a virion and transmitted between hosts. A minus-strand virus is one in which the minus strand is packaged and transmitted. A double-strand virus is one in which double-strand RNA is packaged and transmitted. Our aim is to understand what determines which of these strategies is most successful evolutionarily. As all these strategies exist in nature, we want to explain why different strategies are selected in different kinds of virus.

The diversity of plus-strand viruses is the highest [2], which suggests that double-strand and minus-strand viruses may have arisen from plus-strand ancestors. Double-strand viruses share several common features with plus-strand viruses [3], which suggests that they may have evolved from plus-strand viruses that used a double strand as an intermediate stage in replication. Minus-strand viruses are the least diverse [4], suggesting a more recent origin. However, they are sufficiently different from plus-strand viruses that the pathway of origin of minus-strand viruses is unclear. Phylogenetic studies of viruses are difficult due to rapid rates of sequence evolution and few genes shared across all virus groups. However, recent phylogenetic studies of polymerase protein sequences [5,6] suggest that double-strand viruses may have arisen several times from within plus-strand viruses, and that minus-strand viruses are monophyletic, having arisen only once.

Initially, the plus-strand strategy seems to be the most logical. A virion containing a plus strand can immediately be translated by host ribosomes. This produces virally encoded proteins, including an RNA-dependent RNA polymerase, RdRp, which is necessary for replicating the viral RNA. A virion containing only a negative strand would not be infectious because it could not be translated or replicated if no plus strands were present. However, a virion containing a minus strand can still be useful if it infects the same cell as a plus virion, and producing additional minus-strand virions might be advantageous for competition between viruses if the multiplicity of infection is high.

The reason for the existence of purely minus-strand viruses does not seem obvious at the outset. For a minus-strand virion to be infectious, it must also contain a polymerase in the capsid. If a polymerase is present, then the first step on entering a new cell is to copy the minus strand to make a plus, after which translation and further replication are possible. But if the capsids do contain a polymerase, then both the plus and minus virions should be infectious. In this case, why should minus strands be packaged into virions rather than plus strands or both?

The primary difference between the two types of strands is that a plus strand encodes genes and a minus strand does not. The origin of the symmetry breaking between the roles of template and catalyst strands has been studied previously in the context of primordial genomes in the RNA World [7,8,9]. Here, however, we are interested in viruses that are parasites of modern cells with ribosomes, and the questions are different from those in the RNA World. We assume that the difference between strands exists from the outset, with genes encoded only on the plus strand. We ask questions about the symmetry of packaging and production steps for the two strands. By packaging symmetry, we mean whether the virus should package only plus strands, or only minus strands, or package both kinds of strands into separate particles. By production symmetry, we mean whether the virus should produce equal numbers of plus and minus strands, or if one type of strand should be favored.

Assembly of a virus requires interaction between the RNA and the capsid proteins. Packaging signals along the viral sequence bind specifically to the capsid proteins [10,11], allowing the virus to package its own genome and not the host RNAs that are also in the cell. The virus is free to evolve the presence or absence of packaging signals on the plus or minus strands. In other words, the packaging strategy is under the evolutionary control of the virus and is subject to natural selection. For single-stranded viruses, it is possible to choose to package either the plus or the minus strands, but there also seems no reason why it should not choose to package both, given that both kinds of strands exist in the cell. As far as we know, there are no single-stranded viruses that package both plus and minus strands separately. But this raises the question of why not. Would a strategy that packages only one kind of strand be selected over a strategy that packages both?

The production strategy is also under the evolutionary control of the virus and is subject to natural selection. Replication requires recognition of a template strand by a polymerase. Specific structures in the viral RNAs allow binding to the polymerase and ensure that the polymerase replicates the viral strands and not the RNAs of the host. Such structures have been identified in many viruses [12,13,14]. Separate binding sites are required on plus and minus strands, and these need not be equally efficient. If the polymerase binds more slowly to the plus strand, then minus strands will be used more frequently as templates, and more plus strands will be synthesized. There is a potential advantage of producing more plus strands because only plus strands can be translated. Therefore, we ask whether it is favorable to evolve weaker polymerase binding on one strand in order to increase the ratio of plus to minus strands. When there is a double-strand replicative form, it is also possible to adjust the ratio of plus to minus single strands that is synthesized from a double strand, as occurs, for example, in bacteriophage ϕ6 [15].

**Figure 1:**
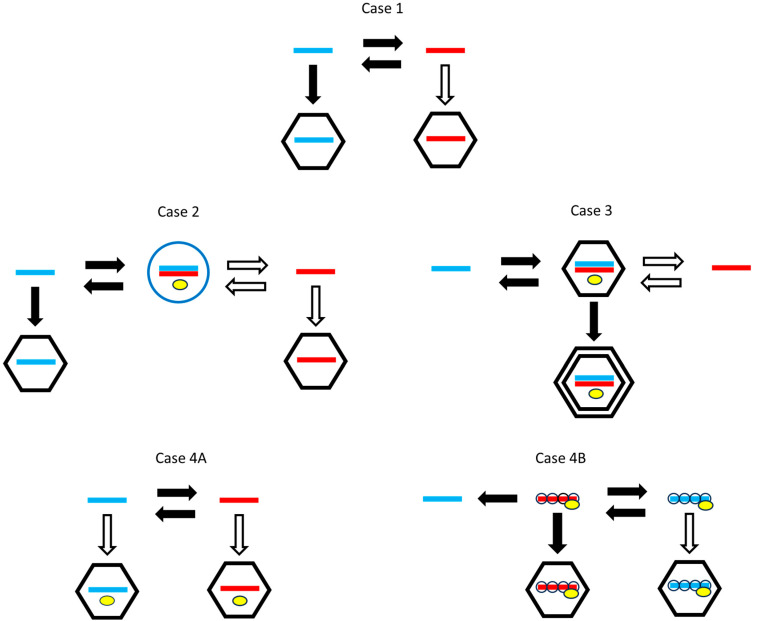
Possible life cycles for RNA viruses. Plus strands are blue. Minus strands are red. Horizontal arrows indicate strand replication. Vertical arrows indicate packaging. Black arrows indicate necessary steps for all viruses following this life cycle and that are seen in real viruses. White arrows are optional steps that are performed by some but not all of the possible strategies and are not necessarily seen in real viruses. Yellow symbols indicate the RdRp when this is incorporated in the capsid or replication complex. Case 1: Single-stranded viruses with alternating replication and polymerase not included in the virion. Case 2: Single-stranded viruses with a double-stranded replicative form. The double strand is enclosed in a membrane-associated vesicle (blue circle). Case 3: Double-stranded viruses. Replication occurs in an inner-core protein layer which is later included in the outer layer of the capsid. Case 4A: Single-strand viruses with alternating replication and a polymerase included in the virion. Case 4B: Same as 4A, with the added feature that genomic strands are covered by a nucleoprotein, and separate translatable plus strands are not coated by the nucleoprotein.

Viruses differ in many factors of the life cycle in addition to the packaging and production differences that interest us. Therefore, it is necessary to compare related viruses that work in the same way but differ in packaging or production strategies. Figure 1 shows several different life cycles found in real viruses. We will consider the evolution of packaging and production strategies in each of these cases.

Case 1—Single-strand viruses with alternating replication that do not include a polymerase in the virion. By alternating replication, we mean that a plus strand is used as a template to make a minus strand, and a minus strand is used as a template to make a plus. This applies to simple bacteriophages like Qβ, where it is known that the polymerase has separate exit channels for the template and newly synthesized strands, so that the new strand is kept separate from the template. During replication, the elongation factor EF-Tu, bound to the Qβ polymerase, facilitates the separation of the growing strand and the template [16]. Qβ does not package a polymerase in the virion, so the polymerase must be synthesized from a plus strand when the virus enters a new cell. We will denote possible strategies as P (package only plus strands), M (package only minus strands), and B (package both strands separately). For this case, packaging plus strands is necessary (a black arrow in Figure 1) and packaging minus strands is optional (white arrow). An M strategy would not be viable, because a minus virion would not be infectious when there is no polymerase in the capsid. However, a B strategy would be viable, and it might be beneficial when multiplicity of infection is high, if it produces a larger total number of virions than the P strategy. The main question in Case 1 is whether the P strategy beats the B. We expect that it should do so, given the prevalence of plus-strand viruses and the absence of B viruses in nature. We also ask whether the production rate of plus strands should be higher than that of minus strands.

Case 2—Single-strand viruses with a double-strand replicative form that do not include a polymerase in the virion. A replicative form (RF) is a double strand that occurs in the life cycles of many plus-strand viruses. When a single plus strand is used as a template, the resulting minus strand remains attached to the template as a double strand. The minus strand within the double strand can then be used as a template for new single plus strands, via a strand-displacement mechanism. Most plus-strand viruses work in this way. For instance, coronaviruses like SARS and mouse hepatitis virus and alphaviruses like Semliki Forest virus use minus strands present in the RF as templates to synthesize the plus strands [17,18]. The RF is usually housed in an invaginated vesicle known as a spherule, formed on one of the internal membranes of the host cell [2,19,20]. The replication step occurs inside the spherule and single plus strands are released from the spherules into the cytoplasm, where they can either be translated, packaged into virions, or form new RFs.

Spherule formation requires the presence of virally encoded proteins that induce the invagination in the host membrane. For example, a viral factor, protein A, is required for Flock House virus to form the invagination and successfully replicate itself [21,22], and brome mosaic virus (BMV) needs to express replication factor 1a that can induce spherule formation [23]. For hepatitis C virus, the plus strand must be translated to produce large amounts of structural and non-structural proteins prior to the formation of the RF for RNA synthesis [24]. Replication also requires expression of RNA polymerase. Hence, the first step in a new cell must be translation, which means that a minus-strand virion is not infectious when alone (as also with Case 1).

In Case 2, we can ask what is the optimal ratio of synthesis of plus and minus strands from the RF. It may be unnecessary to synthesize any single minus strands at all; for example, in tombusvirus [25], it was found that minus strands were always part of double strands, and single minus strands were not detected. If minus strands are synthesized from the RF, we can ask whether both strands should be packaged or only the plus strands. If only plus strands are synthesized, only plus strands can be transmitted in virions.

Case 3—Double-strand viruses. In this case, virions contain double-strand RNA, and have an inner-core layer of proteins surrounded by an outer layer. When a virion enters the cell, the outer layer disassembles, but the core layer remains around the RNA. For example, in reovirus, the core entry into the cell cytoplasm is mediated by proteolysis of the outer layer, followed by conformational rearrangements of membrane-penetration proteins [26]. The core contains a polymerase. Single strands are copied from the double strand and released from the core into the cytoplasm. Single plus strands are translated and are eventually assembled into new core particles and complete virions.

Double-strand viruses have similarities with plus-strand viruses that have a double-strand RF [3]. In both cases, there is a double-strand form that is used to synthesize single plus strands. Reovirus σNS and μNS proteins induce changes in endoplasmic reticulum membranes, leading to spherule formation [27] in a similar way to plus-strand viruses. In both cases, the double strand is kept away from host enzymes that would degrade double-strand RNA by enclosing it in a spherule or in the inner core of the virion. Formation of the spherule and formation of the inner core both require virally encoded proteins. In the plus-strand case, the proteins in the spherule are separate from those in the virion. In the double-strand case, the inner-core proteins are surrounded by the outer-layer proteins when the complete virion is formed. In both cases, it appears that only single plus strands are synthesized from the double strand. Questions to be asked in Case 3 are what the optimal ratio of synthesis of plus to minus strands from the double strand is, and whether it is necessary to synthesize any single minus strands at all.

Case 4—Single-strand viruses with alternating replication that contain a polymerase in the virion. Naturally occurring minus-strand viruses, such as influenza virus, Ebola virus, rabies virus, and human respiratory syncytial virus all fit this case [28,29,30,31]. By including a polymerase in the virion, a minus-strand virion becomes infectious. The first step upon entering a cell is the synthesis of a plus strand that can then be translated. An M strategy that packages only minus strands is a viable strategy in this case (in contrast to Cases 1–3). However, if a plus-strand virion is also infectious, P and B strategies are also viable. The question to be addressed in Case 4 is under what circumstances an M strategy is selected over a P or B strategy. We find here that in a model in which both plus and minus virions are infectious (Case 4A), the B strategy outcompetes both P and M. Hence, the inclusion of the polymerase in the virion is necessary but not sufficient to explain why minus-strand viruses evolve.

Case 4B shows an additional factor present in the life histories of real minus-strand viruses. The replicable plus and minus strands of minus-strand viruses are coated by a nucleoprotein along their whole length, and are also associated with a polymerase, in the form of a ribonucleoprotein complex, e.g., in influenza A [32]. The polymerase is able to copy templates which have nucleoprotein attached [33,34] without complete dissociation of the nucleoprotein. Newly synthesized replicable strands are coated with nucleoprotein as they form [35], and thus protected from degradation by host ribonucleases. Alternating replication occurs between the replicable (coated) plus and minus strands. However, the coated form of the plus strand cannot be translated by the ribosome. Therefore, a second kind of translatable plus strand must be made which can function as an mRNA. The translatable plus strand is not coated by the nucleoprotein and cannot be copied by the polymerase. There is a distinction between replication, which creates a new replicable strand, and transcription, which creates a translatable strand. The transcription step can occur as the first step after a minus virion enters the cell. However, the replication step requires nucleoproteins, and can only occur after translation of these proteins from the mRNA. The nucleoproteins cannot be synthesized without a translatable strand, and the translatable strand can only be transcribed from a minus strand. A plus-strand virion is not infectious because it can neither be translated nor replicated, which is contrary to the assumption made in Case 4A. Given that only the minus-strand virion is infectious in Case 4B, the possible strategies are M and B, and we show below that an M strategy can be selected over a B strategy in this case, which explains the existence of minus-strand viruses.

In summary, the goals of this work are to use evolutionary models of the life cycles of RNA viruses in order to explain why separate plus-strand, minus-strand, and double-stranded viruses exist in nature and can all be successful strategies in different cases.

## 2. Methods

### 2.1. Replication Steps inside One Cell

For each of the cases in Figure 1, we use a stochastic model to define the individual steps involved in virus replication inside one cell. These steps are shown for case 1 in Table 1. This model applies to simple viruses such as Qβ, which have alternating plus and minus strand replication and do not include a polymerase in the capsid. We use the following notation. $X^{+}$ and $X^{-}$ are single plus or minus strands. $V^{+}$ and $V^{-}$ are virions containing plus or minus strands. R is an RNA-dependent RNA polymerase, and C is capsid proteins. Rate constants for replication, translation, and virion assembly are denoted k, v, and a. The steps in the viral life history are shown in Table 1.

Steps 1 and 2 are the replication of viral RNA. A plus is produced from a minus, and a minus from a plus. The rate depends on the polymerization rate constant *k*, the concentration of the template strand, and the concentration of polymerases. We consider a fixed size of host cell, so we use the numbers of molecules per cell, $X^{+},\;X^{-},\;R,$ etc., in place of concentrations. We expect that the polymerization rate is proportional to the number of polymerases when this number is small, and that it saturates when this number is large, because the speed at which a polymerase moves along the template is independent of the number of polymerases. We define a linear saturating function $f_{1}\left(z\right)=z/(1+z)$, and suppose that the rate depends on $f_{1}\left(R/R_{0}\right)$, where $R_{0}$ is the value at which the rate reaches half its maximum. We set $R_{0}=5$ throughout this paper.

Steps 3 and 4 are translation of the plus strand by the host ribosomes to give polymerases and capsid proteins. Multiple capsid proteins are required for each virus particle (e.g., 60 for a T1 icosahedral capsid), whereas only one polymerase is required per virus strand. By C=1, we denote a quota of capsid proteins that is sufficient to make one complete capsid, whereas R=1 denotes a single polymerase. We deal with synthesis of a quota of capsid proteins as a single step because having 60 separate steps for individual proteins would slow down the simulation a great deal. By setting the translation rate *v* to be the same for a polymerase and a quota of capsids in 3 and 4, we make the synthesis of individual capsid proteins much faster, which is necessary to avoid producing a large excess of polymerases. We have previously used a very similar model to study the evolution of bipartite viruses from monopartite viruses [36] and have dealt with translation of the capsid proteins in the same way.

Steps 5 and 6 are the assembly of complete viruses. This requires one RNA strand and one quota of capsid proteins. The assembly rate depends on the rate constant a and is proportional to the number of viral RNAs. We assume that the assembly rate is non-linear in the capsid protein number because it requires nucleation of the capsid by several separate capsid proteins. We define a non-linear saturating function $f_{4}\left(z\right)=z^{4}/(1+z^{4})$, and suppose that the rate depends on $f_{4}\left(C/C_{0}\right)$, where $C_{0}$ is the value at which the rate reaches half its maximum. We set $C_{0}=20$ throughout this paper. For $C<C_{0}$, assembly will be very slow relative to translation and replication, which allows virus strands to multiply to a fairly large number before they begin to be assembled. In our previous work [36], we found that if the rate of assembly is linear in the capsid protein concentration, rapid virion assembly occurs when there are still few viral RNAs, which prevents effective replication of the RNA. Therefore, we use the non-linear saturating function in the current work. We consider two principal strategies: B packages plus and minus strands with equal assembly rate, and P packages only plus strands; therefore, the rate of step 6 is zero for P.

### 2.2. Stochastic Simulation Methods for Single Cells

Beginning with a single virion infecting a cell, or a small number of coinfecting virions, we follow the number of strands and virions produced in the cell over time, as in our previous work with bipartite viruses [36]. We use the Gillespie algorithm [37] to simulate the events in Table 1. Given the numbers of each kind of strand and protein, we know the rates of each possible event. One event is chosen at random with a probability proportional to its rate. The numbers of molecules are then changed due to the occurrence of this one event. The mean time for an event to occur is $1/R_{tot}$, where $R_{tot}$ is the sum of the rates of all possible processes. The time δt until the event occurs has an exponential distribution $P\left(\delta t\right)=R_{tot}exp(-R_{tot}\delta t)$. The time *t* since the onset of the infection is advanced by δt at each step of the program.

A cell infected with a $V^{+}$ virion begins with a single $X^{+}$ strand. Translation of this strand is possible immediately, which produces polymerases, which then allows replication to occur. A cell infected by a single $V^{-}$ would begin with a single $X^{-}$, which cannot be translated or replicated. However, a cell with a $V^{+}$ and a $V^{-}$ would begin with one strand of each type, and replication would proceed.

We assume that the virus stops multiplying when it exhausts the resources of the host cell. There is a protein resource limit such that the maximum number of quotas of capsid proteins that can be produced per cell is $C_{max}$. The number of quotas of capsid proteins that have been synthesized at any point in time is the number incorporated into virions plus the number of unassembled capsids, ${C_{tot}=V}^{+}+V^{-}+C$. Translation steps 3 and 4 occur as in Table 1 as long as $C_{tot}<C_{max}$, but we set these rates to zero once $C_{tot}=C_{max}$. We also include a nucleic acid resource limit such that the maximum number of strands that can be synthesized is $S_{max}$. The total number of strands that have been synthesized at any point in time is ${S_{tot}=X^{+}+X^{-}+V}^{+}+V^{-}$. Replication steps 1 and 2 occur as in Table 1 as long as $S_{tot}<S_{max}$, but we set these rates to zero once $S_{tot}=S_{max}$. Assembly steps 5 and 6 continue to occur after the resource limits are reached, because assembly can occur using strands and capsid proteins that have already been synthesized.

### 2.3. Methods for Simulating Transmission of Viruses in a Host Population

For transmission in a population of host cells, we use two methods that we call the *λ* method and the *α* method. In the λ method, λ is the mean number of virions that infect a host cell (the MOI). The probability that a cell is infected by *n* virions is a Poisson distribution $P\left(n\right)=e^{-\lambda }\lambda^{n}/n!$. The frequencies of virions of type $V_{i}^{j}$ are $p_{i}^{j}$, where *i* is a strategy and *j* is + or −. For each newly infected cell, the *n* virions infecting that cell are chosen independently with probabilities $p_{i}^{j}$. The number *n* and the types of infecting particles are chosen independently for each cell. We consider a fixed population size of 1000 cells, and we only consider cells with at least one infection, *n ≥* 1. Hence, the probability of beginning with *n* virions in a cell, given that there is at least one, is $P\prime \left(n\right)=P(n)/(1-e^{-\lambda })$, and the mean number of virions per cell, given that there is at least one, is $\lambda \prime =\lambda /(1-e^{-\lambda })$, which tends to 1 when λ is close to 0, and tends to λ when λ is large. The probability of more than one virion infecting a cell is $Q=(1-e^{-\lambda }-\lambda e^{-\lambda })/(1-e^{-\lambda })$, which tends to 0 when λ is small and to 1 when λ is large.

Each cell is run for a time *t* = 100, at which point virions are released. We sum up the total number of virions of each type produced by the whole population, and calculate the output virion frequencies as $p_{i}^{j}=V_{i}^{j}/\sum_{ij}V_{i}^{j}$. These frequencies are then used for the input to the next generation. After many generations, the frequencies converge to stationary values. We run the simulation for 1000 generations. The frequencies are averaged over the second half of the simulation after a stationary state is reached. Note that in the transmission simulations, we consider multiple cell generations, and we allow any number of simultaneous infections per cell, as determined by the Poisson distribution, whereas in the single-cell simulations in the previous section, we considered only one cell generation, and we simply calculated the mean numbers of viruses produced per cell. In the single-cell simulations, we only consider examples of one or two virions infecting a cell, but cases where *n* > 2 can occur in the transmission simulations.

When using the λ method, we are assuming that the number of new infections is always sufficient for the virus to survive. As λ→0, λ′→1, and the virus that survives in this limit is the one that produces the largest number of infectious virions when it is alone in a cell. There is always one virus that survives when using this method.

In our previous studies of bipartite viruses [36], we used an alternative method, which we call the α method. In the α method, we assume that the MOI is proportional to the number of viruses released at the previous generation. The mean number of virions of type *ij* produced per cell at the previous generation is $\langle V_{i}^{j}\rangle$. The maximum possible number is $C_{max}$. Therefore, we suppose that the mean number of virions of this type that infect a cell at the current generation is $\lambda_{i}^{j}=\langle V_{i}^{j}\rangle /C_{max}$. In this method, α is the transmissibility rate of the virus. The number of virions of type *ij* that infect a cell, $n_{i}^{j}$, is chosen separately for each type with a Poisson distribution having mean $\lambda_{i}^{j}$. Hence, each type has its own MOI, and the MOI is not fixed. In this method, there are some cells that have no infections, and the fraction of infected cells is an increasing function of α. There is a minimum transmissibility, $\alpha_{min}$, below which a virus dies out because the transmission rate is too small. The virus that survives at a low transmission rate is the one that has the lowest $\alpha_{min}$, which is the one that produces the largest number of infectious virions when it is alone in a cell.

These two methods differ only slightly. The α method assumes that there is random mixing of susceptible hosts with the viruses released by the previous host generation, hence the MOI for each generation depends on the fraction of infected cells at the previous generation. However, in the λ method, the MOI is fixed independently of the fraction of the population infected. This might be the case for a virus that spreads locally by close contacts, in which case the MOI is determined by how likely a virus is to spread by a single contact, which is independent of the fraction of the population that is infected. We will give examples of both methods below. In practice, the conclusions of the two methods are very similar. When the MOI λ, or the transmissibility α, is low, the virus that produces the largest number of infectious particles when alone will be selected. When λ or α is high, the virus that produces the largest total number of virions in multiply infected cells will be selected.

## 3. Results

### 3.1. Model 1—Single-Strand Viruses with Alternating Replication and No Polymerase in the Capsid

The steps in model 1 were described in Section 2.1 above. Before looking at the simulations of this model, we consider the following simple argument that predicts the essential behaviour of the model. We call this the replication-before-packaging (RBP) approximation, because it assumes that RNA replication is completed before the packaging of strands into virions begins.

Consider a cell infected by a single B virion. The virus produces an equal number of plus and minus strands until the limit $S_{max}$ is reached; therefore, the number of strands of each type produced is $S_{B}^{+}=S_{B}^{-}=S_{max}/2$. Capsid proteins are produced up to the limit $C_{max}$. Throughout this paper, we will keep $C_{max}=100$, and we will vary $S_{max}$ relative to this. If $S_{max}=100$, then $S_{B}^{+}=S_{B}^{-}=50$. There are sufficient capsids to accommodate all the RNA strands; therefore, the numbers of virions of each type produced are $V_{B}^{+}=V_{B}^{-}=50$. This is shown in the top line of Table 2. If $S_{max}=150$, then $S_{B}^{+}=S_{B}^{-}=75$, and if $S_{max}=200$, then $S_{B}^{+}=S_{B}^{-}=100$. In both these cases, the number of strands exceeds the number of available capsids. But the B strategy packages both types of strands at an equal rate; therefore, the numbers of virions produced is $V_{B}^{+}=V_{B}^{-}=50$ in all cases.

Now consider a cell infected by a single P virion (lines 4–6 of Table 2). The numbers of strands produced for the three values of $S_{max}$ are the same as for the B virus. However, the P strategy assembles only the plus strands. With $C_{max}=100$, there are sufficient capsids to package all the plus strands in all three cases. The number of plus virions $V_{P}^{+}$ is therefore 50, 75, and 100, whereas the number of $V_{B}^{+}$ virions is always 50. Only the plus strands are infectious in Case 1. When $S_{max}>C_{max}$, the number of infectious virions produced by P is more than that produced by B. We argue that this is the primary reason why plus-strand viruses are successful in nature and B viruses are not seen. If RNA strands are in excess over the number of available capsids, then by choosing not to package the minus strands, a P virus produces an increased number of infectious virions.

However, if $S_{max}=C_{max}$, then P has no advantage, because $V_{P}^{+}=V_{B}^{+}$. Furthermore, B produces additional virions with negative strands, $V_{B}^{-}$, which are not produced by P. The $V_{B}^{-}$ virions are not infectious when alone in a cell; however, when the multiplicity of infection (MOI) is higher, it is possible that a negative virion will infect the same cell as a positive virion, in which case replication can proceed from the negative strand. At high MOI, a negative virion is better than no virion at all. In particular, if B and P are in competition in the same host population, then there is a significant probability that a $V_{B}^{-}$ will infect the same cell as a $V_{P}^{+}$, in which case the negative B strand is able to replicate because of the presence of the positive P strand. There is thus a significant benefit to producing additional negative virions when MOI is high. If the MOI is very high, almost all the negative virions will infect cells that also contain positive virions. In this case, the strategy that produces the largest total number of virions should be best, even if half these virions would be non-infectious if they were alone in a cell. From Table 2, when $S_{max}<2C_{max}$, the total number of B virions, $V_{B}^{+}+V_{B}^{-}$, is greater than the number of P virions, $V_{P}^{+}$. Thus, we expect B to do well at a high MOI.

To further understand the case where B and P infect the same cell, we consider cells that begin from a single strand of each type in one cell (lines 7–9 of Table 2). We suppose that it does not matter whether the B strand is plus or minus, because the P strand is always plus. In this case, four types of strands are produced in equal numbers, but only three types of strands are packaged to virions. Therefore, $V_{B}^{+}=V_{B}^{-}=V_{P}^{+}$, and the total number of B virions is twice the number of P virions.

The conclusion from the RBP approximation is that if $S_{max}>C_{max}$, P has an advantage at a low MOI, but B should have an advantage at a high enough MOI. The value of MOI required for B to win should be higher when $S_{max}$ is larger. When $S_{max}=C_{max}$, P has no advantage and we might expect B to win even for very low MOI. However, this is only a crude approximation, because it is not true that packaging begins only after RNA replication has finished. We therefore consider simulations of the stochastic model in which these processes overlap in time.

### 3.2. Model 1—Simulations of the Stochastic Model in a Single Cell

**Figure 2:**
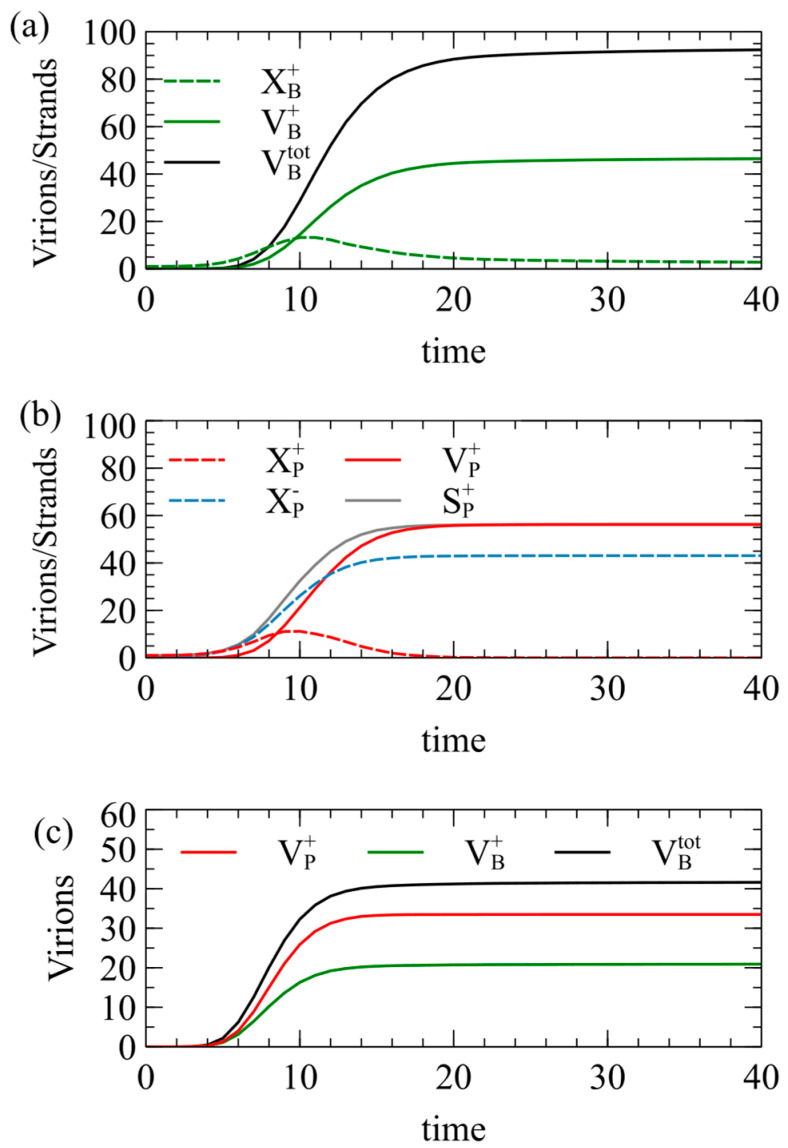
Simulations of model 1 for the case of $S_{max}=C_{max}=100$. Mean numbers of strands and virions formed per cell as a function of time, beginning from (**a**) a single B strand, (**b**) a single P strand, and (**c**) one B and one P.

The following standard parameters are used throughout this paper, unless otherwise specified: $a=k=v=1,R_{0}=5,\;C_{0}=20,\;C_{max}=100.$ Initially, we also set $S_{max}=100$. Figure 2 shows the mean number of viruses produced per cell as a function of time, averaged over a population of 1000 independent cells.

In Figure 2a, each cell begins with a single $X_{B}^{+}$ strand; in Figure 2b, each cell begins with a single $X_{p}^{+}$ strand; and in in Figure 2c, each cell begins with one $X_{B}^{+}$ and one $X_{P}^{+}$. In Figure 2a, $V_{B}^{+}$ approaches 50 at long times, but is slightly lower than this, because the maximum possible number of capsids is not always formed in every cell. The number of negative virions $V_{B}^{-}$ is almost exactly equal to $V_{B}^{+}$, and is therefore not shown. The number of single strands $X_{B}^{+}$ increases at first, and then decreases as the strands are packaged to virions. $X_{B}^{-}$ is almost exactly equal to $X_{B}^{+}$. The number of single strands becomes very low at long times because almost all of them are packaged into virions. The total number of virions $V_{B}^{tot}=V_{B}^{+}+V_{B}^{-}$ becomes close to 100 as expected, but it is slightly less than 100, even for very long times, because in a small fraction of cells, all the RNA strands become packaged before the limit of $S_{max}$ strands has been reached, or before the limit of $C_{max}$ capsids has been reached.

In Figure 2b, for the P strategy, the number of single plus strands $X_{P}^{+}$ increases and then decreases to almost zero, but the number of single minus strands $X_{P}^{-}$ increases to a large constant value because minus strands are not packaged. The number of plus virions $V_{P}^{+}$ reaches 56.2 at long times, which is significantly higher than the number $S_{max}/2=50$ expected from the RBP approximation. We have assumed that plus and minus strands are produced at equal rates, so how can $V_{P}^{+}$ be more than 50? The answer is that the packaging of plus strands begins before RNA replication is complete. Plus and minus strands start to be synthesized at the same rate, but some of the plus strands are removed by packaging. This leaves more minus than plus strands remaining in the cell. As a result of the larger number of minus templates, the rate of synthesis of new plus strands is higher. By the time that the total number of strands synthesized reaches the limit of $S_{max}$, the number of plus strands that have been synthesized is significantly greater than $S_{max}/2$. Figure 2b shows the number of plus strands synthesized, $S_{P}^{+}=X_{P}^{+}+V_{P}^{+}$, which is noticeably above the number of minus strands, $X_{P}^{-}$. Almost all the plus strands are packaged; hence the final number of plus virions is greater than $S_{max}/2$.

Figure 2c shows results in which B and P are in the same cell. In this case, $V_{B}^{+}$ and $V_{B}^{-}$ are equal, but $V_{P}^{+}$ is significantly greater than either of these. This is for the same reason: by not packaging the $X_{P}^{-}$ strands, more negative templates remain, and the rate of production of new $X_{P}^{+}$ strands is increased above that of $X_{B}^{+}$ strands.

The numbers of strands and virions produced for simulations of model 1 are given in Table 3. When $S_{max}=100$, $V_{B}^{+}$ is significantly less than 50 when B is alone, whereas $V_{P}^{+}$ is significantly more than 50 when P is alone. This means that P has a definite advantage at low MOI even when $S_{max}=100$, which was not the case according to the RBP approximation. For $S_{max}$ = 150 and 200, the advantage of P increases with $S_{max}$ in a similar way to the RBP approximation. For the case where B and P are in the same cell, $V_{P}^{+}$ is higher than $V_{B}^{+}$ and $V_{B}^{-}$ for all three values of $S_{max}$, whereas these three are all the same according to the RBP approximation. The conclusion is that P should have a definite advantage over B at low MOI for all values of $S_{max}$, and that the advantage of P is larger than predicted by the RBP approximation. However, there should still be an advantage of B at a high enough MOI. To understand how this will affect viral evolution, we need to conduct simulations of transmission of viruses within populations.

### 3.3. Model 1—Transmission over Time

**Figure 3:**
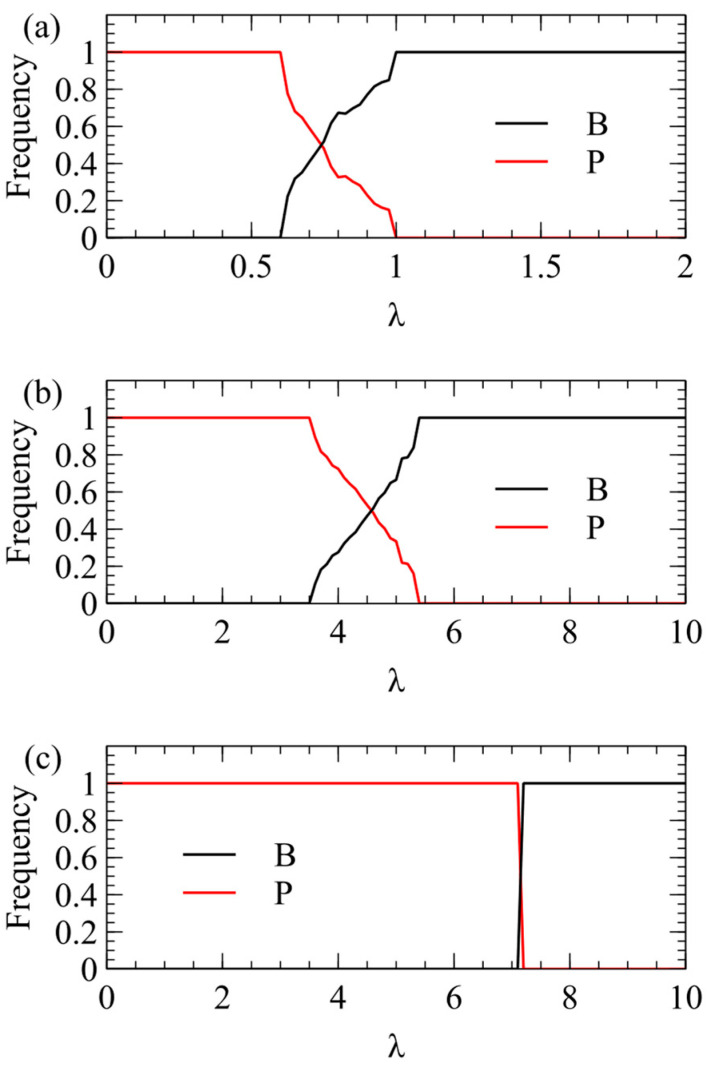
Mean frequency of B and P in the virus population in simulations using the λ method of transmission. (**a**) $S_{max}=100$. (**b**) $S_{max}=150$. (**c**) $S_{max}=200$.

We now consider transmission simulations for competition between B and P viruses using the λ method. These simulations use the same parameters as Figure 2: $a=k=v=1,R_{0}=5,\;C_{0}=20,\;C_{max}=100.$ We begin with four virion types having equal frequencies $p_{i}^{j}=0.25,$ and measure the average frequencies over multiple generations after a steady state has been reached. In Figure 3a, we plot the total virion frequency for each virus in the steady state $p_{i}=p_{i}^{+}+p_{i}^{-}$. As we expect from the single-cell simulations, P wins at low λ, and therefore the frequency of P tends to 1. At higher λ, there is a region where both strategies coexist, and at even higher λ, the frequency of B goes to 1. In Figure 3a, where $S_{max}=100$, P wins only for quite low λ, and B outcompetes P already for λ > 1. In Figure 3b, where $S_{max}=150$, the transition is shifted to higher λ (note change of scale). B outcompetes P for λ > 5.5. In Figure 3c, where $S_{max}=200$, the transition is shifted to even higher λ. B outcompetes P for λ > 7.

**Figure 4:**
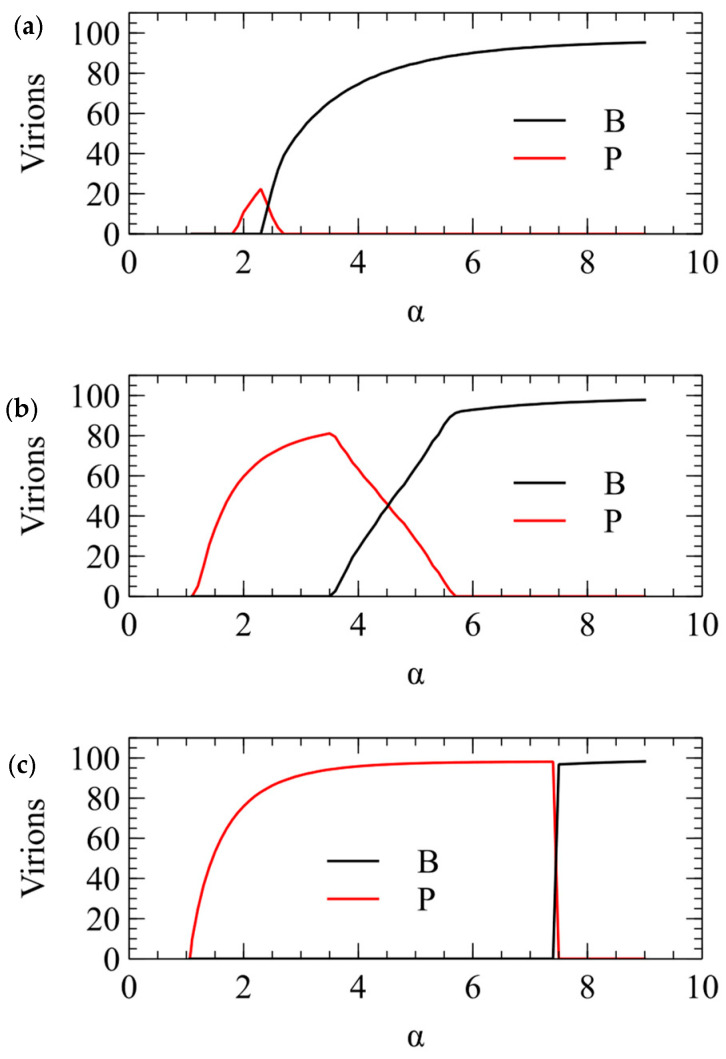
Mean number of P and B virions produced per cell in simulations using the α method for transmission. (**a**) $S_{max}=100$. (**b**) $S_{max}=150$. (**c**) $S_{max}=200$.

We now show the same three cases with the α method of transmission. With this method, there is a minimum transmission rate below which no virus survives. The virus that survives at the smallest α is the one that produces the largest number of infectious virions when alone in a cell. This is always P, for all three values of $S_{max}$. For $S_{max}=100$, there is only a narrow range of transmission rate where P beats B, but for higher $S_{max}$, the range of α is much larger. The conclusions from the λ and α methods are the same, and Figure 3 and Figure 4 are two different ways of illustrating the same outcome.

In summary, model 1 explains the frequent observation of plus-strand viruses in nature, and shows that the advantage of plus-strand viruses is largest if viral RNAs are produced in excess over the number of capsids that can be produced by a cell. If this is true, a potential B virus would only be successful at a very high MOI that is likely to be too high to be achieved consistently by real viruses. This explains why B viruses are not observed in nature.

### 3.4. Model 1—Strategies with Biased Replication Rates

We now ask whether it is beneficial to adjust the rates of production of plus and minus strands so that the proportion of infectious plus strands is increased. We assume that there is a maximum rate k at which polymerases can operate, and that plus strands are produced at this maximum rate because the polymerase has already evolved to be as fast as possible. The proportion of plus strands can be increased by decreasing the rate of minus-strand production, but not by increasing the rate of plus-strand production, because k is already at the maximum. We now consider a strategy Px, for which the rate of production of minus strands is reduced to xk, with x≤1, but the rate for plus strands is still *k.* When x<1, there is an increased proportion of plus strands, but this comes at a cost of reducing the overall replication rate of the virus. We consider *x* = 0.7 as an example.

**Figure 5:**
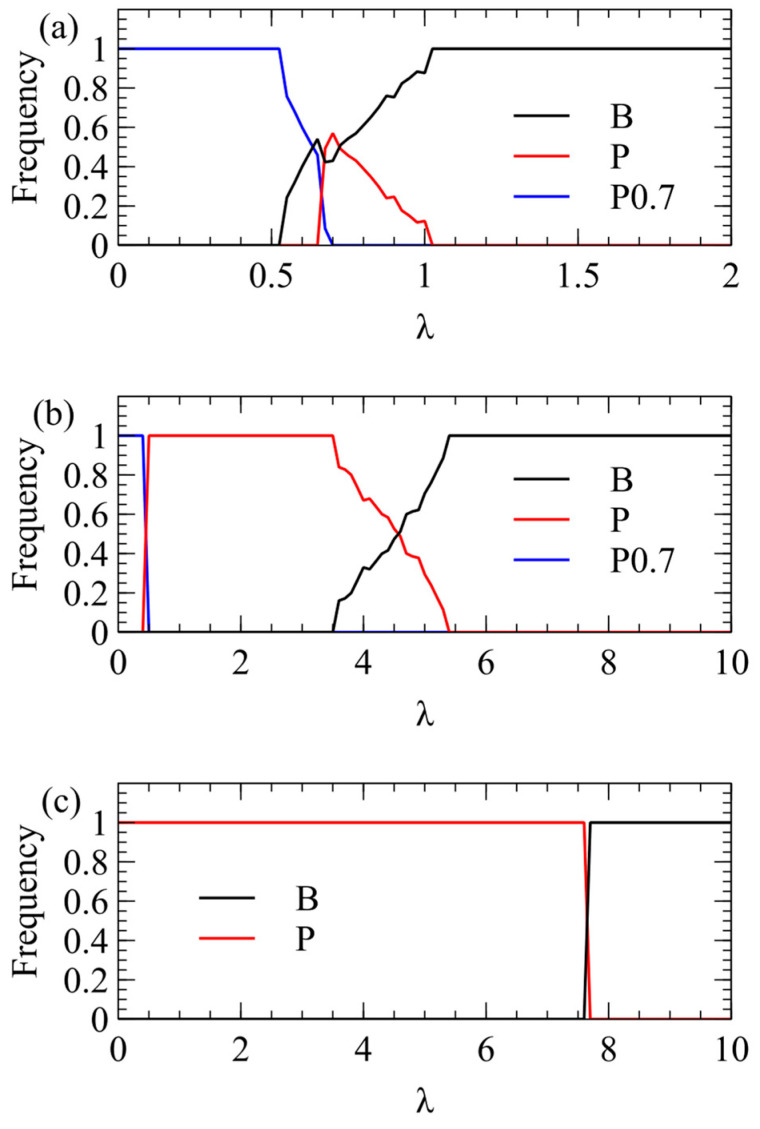
Competition between the biased P0.7 strategy and the original P and B. (**a**) $S_{max}=100$. (**b**) $S_{max}=150$. (**c**) $S_{max}=200$.

Figure 5 shows simulations with P0.7, together with the original unbiased P and B strategy, all with equal frequency initially. For $S_{max}=100$ (Figure 5a), P0.7 wins only for λ<0.5. There is a small range in which P coexists with other strategies, and B wins for λ > 1. For $S_{max}=150$ (Figure 5b), P0.7 again wins only for λ<0.5. The competition between P and B is very similar to Figure 3b. For $S_{max}=200$ (Figure 5c), P0.7 is always defeated by the unbiased P, and P defeats B up to very high λ, exactly as in Figure 3c.

The conclusion is that strategies with x<1 are favoured only if $S_{max}$ is low and if λ is very low. These do not seem likely parameter ranges for real viruses. The fact that we widely observe plus-strand viruses and not B viruses suggests that $S_{max}$ is high, and if this is the case, biased Px strategies with x<1 are outcompeted by the unbiased P.

### 3.5. Model 2—Single-Strand Viruses with a Double-Stranded Replicative Form and No Polymerase in the Capsid

This model applies to single-strand viruses that replicate via a double-stranded replicative form (RF) and which do not include a polymerase in the capsid. The notation is as for model 1, with the addition of D as the double stranded RF, and N as a non-structural viral protein that is required to form the RF (or the spherule in which it is found). For this model, there are two decisions to be made: whether to produce both plus and minus strands from the RF or only plus strands, and whether to package both strands or only plus strands. As with model 1, packaging only minus strands would not be viable. We will denote the strategies as BB, produce both strands and package both strands; BP, produce both strands and package only plus; and PP, produce only plus and package only plus.

In Table 4, steps 1 and 2 are the formation of the RF from a single strand. This depends in a non-linear way on the concentration of the *N* proteins, so it only begins after translation has caused sufficient accumulation of *N*. *N* represents any protein that is required to form the spherule which houses the RF (e.g., protein A for Flock House virus [21,22], and factor 1a for brome mosaic virus [23]). We use the function $f_{4}(N/N_{0})$ in an analogous way to the function $f_{4}(C/C_{0})$ for capsid assembly. As formation of the RF must occur before formation of complete capsids, we set $N_{0}<C_{0}$, so that formation of the RF begins at a lower protein concentration. We keep $C_{0}=20$ and set $N_{0}=5$ in these examples. The spherules contain polymerases as well as RNA strands and *N* proteins, so the rates of steps 1 and 2 are proportional to $f_{1}(R/R_{0})$. We suppose that the double strand is formed immediately when the spherule is formed, and therefore we treat formation of the double strand and formation of the spherule as a single step. As formation of the spherule requires N and R proteins, a single $V^{-}$ particle is not infectious because it cannot synthesize these proteins. We have introduced a rate constant b for steps 1 and 2, but in practice we have considered examples where all the rate constants are 1.

Steps 3 and 4 are the synthesis of new single strands from the RF. A polymerase is included in the spherule and is assumed to be located very close to the RNA. Therefore, steps 3 and 4 proceed at a constant rate for each D. The total rate of production of single strands from each double strand is k. For BB and BP, the rates of plus- and minus-strand synthesis are equal to k/2, but for PP, only plus strands are synthesized, so the plus-strand rate is *k* and the minus-strand rate is 0. Single minus strands are possible but not necessary in this model because a single plus carries out all the functions of a single minus. Hence, it is possible to have a PP strategy that produces no minus strands in this model, in contrast to model 1, where minus strands must be produced for alternating replication. Steps 5–9 are translation and virion assembly, similar to model 1. The assembly of negative virions (step 9) occurs only for the BB strategy.

**Figure 6:**
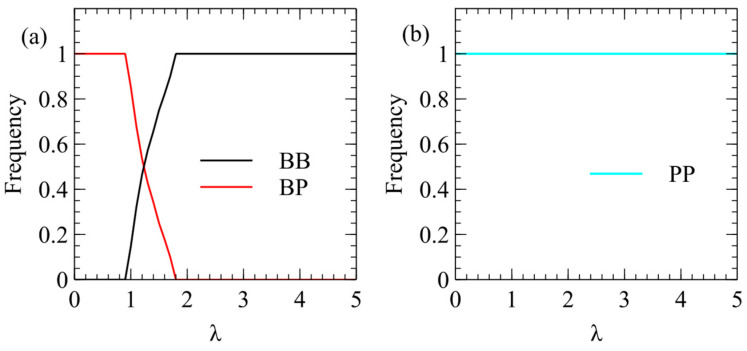
Mean frequencies of strategies in model 2. (**a**) In competition between BP and BB only, BP wins at low λ and BB wins at high λ. (**b**) In competition between PP, BP, and BB, the winning strategy is always PP and the other strategies are always eliminated.

Figure 6 shows mean frequencies of strategies in model 2 with: $a=b=k=v=1,R_{0}=5,\;{N_{0}=5,\;C}_{0}=20,\;C_{max}=100,$ and $S_{max}=200$. In Figure 6a, only BB and BP are included in order to consider the benefits of packaging one or both strands independently of the decision whether to produce both strands. We find that BP wins at low λ and BB wins at high λ. In other words, packaging only plus strands is beneficial at low λ, the same as model 1 (Figure 3). However, in Figure 6b, we begin with three strategies, PP, BP, and BB, and we find that PP always wins for all λ. These results are shown with excess strands over capsids ($S_{max}=200$), but we have also considered the case with $S_{max}=100$, and we find the same result, that PP always wins.

The conclusions of model 2 are simpler than those of model 1. In model 2, single minus strands are not necessary, and the optimal strategy is PP, which produces no minus strands from the double strand. The only minus strands in this model are part of the double-strand RF and there are no single minus strands in the cytoplasm. The majority of plus-strand viruses fit the life cycle of model 2, and this model clearly predicts that plus-strand viruses should outcompete B viruses, as is observed in nature. It should be noted that we have assumed that it is possible to make the rate of synthesis of minus strands from the double strand zero by adjusting the relative rate of binding of the polymerase to the two sides of the double strand, and that this does not prevent the formation of the double strand from the single plus strand.

### 3.6. Model 3—Double-Strand Viruses

Model 3 is for viruses in which double-strand RNA is transmitted. Model 3 (Table 5) is very similar to model 2 (Table 4). In real double-strand viruses, the double strand remains inside the core layer of the capsid while synthesis of new single strands occurs, and the single strands are released to the cytoplasm. Here, D is a double-strand in a core particle, rather than a double strand in a spherule. N proteins are those that make the core layer, rather than those that make the spherule. Steps 1–7 are the same as in model 2. Step 8 is the assembly of the complete virion, $V_{D}$, from the core particle, D. In this model, all strategies transmit double strands, but they can differ in which single strands are present inside the cell. The strategy DB produces both plus and minus strands from the double strand, and the strategy DP produces only plus strands from the double strand.

The results of simulations of this model are very simple: DP beats DB in all cases. Just as for Case 2, it is not necessary to produce any single minus strands. Thus model 3 successfully explains why double-stranded viruses in nature operate in this way.

### 3.7. Models 4A and 4B—Single-Strand Viruses with Alternating Replication and a Polymerase Included in the Capsid

Minus-strand viruses are not possible in models 1–3. To allow the possibility of evolution of minus-strand viruses, there needs to be a polymerase included in the virion. Model 4A in Table 6 considers alternating replication with polymerases in the virions. The packaging steps 5 and 6 now incorporate a polymerase, and steps 1–4 are the same as in model 1. When virions are transmitted in this model, for every $V^{+}$ or $V^{-}$ virion that enters a cell, a polymerase is released in addition to the $X^{+}$ or $X^{-}$ strand. Therefore, both types of virion are infectious.

The results of the simulation of model 4A are very simple. When initiated with a mixture of P, M, and B strategies at equal frequency, B always outcompetes the other strategies at all λ. Therefore, model 4A shows that simply packaging the polymerase in the virion is not sufficient to account for the evolution of minus-strand viruses. When both kinds of virions are infectious, the model predicts that the *B* strategy should be selected, because it produces more virions in total and both kinds of virions are infectious. As this result is not seen in nature, there must be some factor that is missing from model 4A.

Closer examination of the life cycles of real minus-strand viruses reveals what this missing factor is. As described in the introduction, in minus-strand viruses such as influenza [28], Ebola [29], and respiratory syncytial virus [30], replicable plus and minus strands are coated with a nucleoprotein, N. These strands can be replicated but not translated. The existence of separate replicable and translatable strands can explain the fact that the M strategy is selected, as we now show with model 4B in Table 7.

In model 4B, $Y^{-}$ and $Y^{+}$ are the replicable minus and plus strands that are coated with nucleoprotein. We assume that each of these replicable strands is closely associated with its own polymerase, in a ribonucleoprotein complex [28,29,30]. When a $V^{-}$ or $V^{+}$ virion infects a cell, a $Y^{-}$ or $Y^{+}$ strand is released. Step 1 is the transcription of $X^{+}$ from $Y^{-}$. This can occur without additional proteins because there is already a polymerase associated with the $Y^{-}$. We assume a $Y^{+}$ strand cannot be transcribed; hence there is no way to form a non-coated $X^{-}$ strand, which is indeed the case with real minus-strand viruses. However, if we did allow the formation of an $X^{-}$ as a possible step in the model, it would serve no purpose, because an $X^{-}$ would have the wrong sense for translation and it could not be replicated. There is no reason why a virus should evolve a mechanism for synthesis of an $X^{-}$ strand. Therefore, we do not include this possibility in the model.

Steps 2 and 3 are the replication of replicable plus and minus strands to give the complement. This works with either $Y^{-}$ or $Y^{+}$ as a template, but it requires additional nucleoproteins to coat the new strand and an additional polymerase that becomes associated with the new Y strand. Therefore, these steps can only occur after translation has synthesized R and N. The rate of steps 2 and 3 depends on $f_{1}(R/R_{0})$ and $f_{4}(N/N_{0})$ as in earlier models.

Steps 4–6 are the translation of R, N, and C from $X^{+}$. As before, N represents one quota of nucleoprotein sufficient to coat one strand, and C represents one quota of capsid proteins sufficient to form one virion. Steps 7 and 8 are packaging of the $Y^{+}$ and $Y^{-}$ strands into virions. We assume that either of the replicable strands can be packaged, but not the translatable strand $X^{+}$. The possible strategies are either P, M, or B, according to which strands are packaged.

Having introduced the distinction between replicable and translatable strands, only the minus virion $V^{-}$ is infectious. A $V^{+}$ virion is not infectious when alone in a cell because it releases a $Y^{+}$ strand that can neither be translated or replicated in the absence of nucleoproteins. The P strategy is not viable in model 4B, and the competition is between M and B. This is the mirror image of the situation in models 1 and 2, where M was non-infectious and the competition was between P and B.

When we ran simulations of this model with the same rate parameters that we used for all the previous cases ($a=k=v=1,R_{0}=N_{0}=5,\;C_{0}=20,\;C_{max}=200$), we found that M always outcompetes B at all values of λ. Thus, model 4B explains why minus-strand viruses are found when there is a distinction between replicable and translatable strands, but not when there is no such distinction (as in model 4A).

As model 4B seems like a mirror image of model 1, we wondered whether it would be possible to find cases where B outcompetes M at high λ, just as B outcompetes P at high λ in model 1. We found that by reducing the assembly rate, a, relative to the polymerization and translation rates, k and v, there were examples in which M wins at low λ, M and B coexist at intermediate λ, and B wins at high λ, which is equivalent to model 1 (Figure 3). We have not carried out a full investigation of the changing values of these rates because we do not expect it to change the conclusions. The principal result is that M always wins at low λ; hence model 4B successfully explains why minus-strand viruses can be the winning strategy.

## 4. Discussion

### 4.1. Plus-Strand Viruses

Models 1 and 2 consider cases where single strands are transmitted and there is no polymerase in the capsid. Plus-strand viruses are selected in both models. The difference between models 1 and 2 is that in model 1, there is alternating replication between single plus and single minus strands, whereas in model 2, there is a double-stranded RF. In the second case, replication alternates between a single plus strand and a double strand, and the only minus strands are those contained in the double strand. This distinction seems sufficiently important for us to make two separate models. The conclusion from the models is slightly different. Both predict that P wins at low λ, but B can win at high λ only in model 1. Also, we find that PP is the winning strategy in model 2, suggesting that the ratio of plus to minus strands should be strongly biased in viruses that use a RF, and that there may not be any free minus strands in the cytoplasm. However, in model 1, the unbiased P strategy outcompetes biased strategies Px in most cases, suggesting that there should be little strand bias in viruses with alternating replication.

When trying to establish exactly which viruses use a double-stranded RF, we find the literature somewhat confusing. Ahlquist [3] states that plus-strand virus replication is invariably localized to intracellular membranes (i.e., case 2). But we presume that this is only true for viruses of eukaryotic cells and does not apply to bacteriophages like Qβ. The case of Qβ fits the alternating pattern of case 1, without formation of a double strand. The polymerase of Qβ seems to have evolved to separate the template and newly synthesized strands [16]. On the other hand, the bacteriophage MS2, which is somewhat similar to Qβ, is thought to pass through a double-stranded form [38,39]. Thus, the existence of a double-strand form is not exclusive to eukaryotic viruses replicating inside spherules. Conversely, for eukaryotic viruses, there are plus-strand viruses that form spherules which may not contain double strands. For example, minus strands rather than double strands are treated as replicative intermediates in hepatitis C [40,41]. This case still seems close to our model 2, in that minus strands are found only inside the spherules, and plus strands are released from the spherules into the cytoplasm. 

### 4.2. Relationship between Plus-Strand Viruses and Other Virus Types

We have considered cases 1–4 in separate models because the life cycles involved are significantly different. When we look at competition between strategies in any one of these models, the strategies represent variants of a given viral species which differ in only the packaging or production of RNA strands. These things can evolve easily without the need to change the function of viral proteins and without the need to change the life cycle or the host organism. When considering competition between the strategies in each model, we supposed that proteins synthesized by any of the strategies functioned equally well with all the other strategies. For example, a polymerase encoded by one strategy could also replicate strands of the other strategies, and capsid proteins synthesized by one strategy could also package strands of the other strategies. This means that our models focus on the selective differences caused by the packaging and production strategies themselves, which operate on a short timescale among related viruses. Considering evolution on a broader scale would require introduction of proteins that have different functions in different viruses, and changes at this level may be due to rare events that are chance occurrences.

Nevertheless, it is tempting to try to consider pathways by which one form of virus could evolve into another form on a long timescale. Large-scale molecular phylogenies of viruses [5,6] show that plus-strand viruses are the most abundant and most divergent, from which we can hypothesize that plus-strand viruses are the most ancient. This fits with the observation that plus-strand strategies are logically the simplest. It is therefore interesting to consider pathways by which double-strand and minus-strand viruses could have evolved from plus-strand viruses.

The link between double-strand viruses and plus-strand viruses with a double-strand RF is emphasized by Ahlquist [3]. This link appears strong to us, and it is supported by our work here (models 2 and 3). Both groups alternate between single plus strands and double strands. In both models, there is no reason to synthesize single minus strands, so the best strategy is to synthesize only plus strands from the double strand. Double-strand viruses therefore seem to be plus-strand viruses that have evolved to package the double-strand stage in the life cycle instead of the plus-strand stage. Several separate groups of double-strand viruses were found by phylogenetics [5], which suggests that a double-strand life cycle has evolved from a plus-strand life cycle on more than one occasion.

### 4.3. Origin of Minus-Strand Viruses

The evolution of minus-strand viruses is more difficult to understand because it seems to be a rather large jump from an ancestral plus-strand virus to a current form of minus-strand virus. Phylogenetic studies [5,6] suggest this has happened only once. Our models suggest two pathways by which minus-strand viruses could have originated. The first pathway involves a B virus. The possibility of a B virus that packages both single strands in separate virions was included in our models because it seems a logical possibility that could easily occur in a real virus if this strategy were advantageous. For single-strand viruses with no polymerase in the capsid, models 1 and 2 show that P has a large advantage over B if RNA strands are produced in a cell in excess of the number of virions that can be produced. In most cases, the B strategy can win only at very high MOI. However, if a polymerase is included in the capsid, as in model 4A, then minus-strand virions become infectious. The B strategy is the winning strategy in model 4A. This suggests that B viruses are a real possibility, and it does not seem impossible that a real B virus might one day be discovered.

One possible route for the origin of minus-strand viruses is that an ancestral plus-strand virus started to package the polymerase, at which point it became a B virus (as in model 4A), and subsequently evolved the nucleoprotein and the two separate forms of plus strand. At this point, the plus strand was no longer infectious and the virus became a minus-strand virus (model 4B). It can be argued that both these steps might be advantageous. Packaging a polymerase would allow both kinds of virion to be infectious, hence increasing the total number of infectious virions produced. The subsequent evolution of the nucleoproteins might be beneficial as a way of protecting the replicating viral RNAs from ribonucleases in the host cell.

The second possible route for the origin of minus-strand viruses is that firstly a plus-strand virus evolved the nucleoprotein as a means of protection of its RNA inside the cell. Such a virus would still package uncoated plus strands into virions without polymerases, as with other plus-strand viruses, but its replication mechanism would use alternation of coated plus and coated minus strands. In current minus-strand viruses, a polymerase is always associated with the coated strands in a ribonucleoprotein complex. If this situation arose, then it would be a relatively small step to start packaging the ribonucleoprotein complexes, and these would already contain a polymerase. Once this happened, the minus-strand virions would be infectious, and it would no longer be necessary to package uncoated plus strands. This route passes from a plus-strand virus packaging uncoated plus strands without polymerases to a minus-strand virus packaging coated minus strands with polymerases. In this case, there would never be any plus-strand virions with polymerases.

## 5. Conclusions

The existence of different forms of RNA virus is well known, but the reasons for this are not often discussed. Here we have shown that these differences can be explained in terms of evolutionary theory and simple theoretical models. The models studied here highlight the important factors that determine viral replication and packaging strategies and can explain why all the observed forms of RNA virus can be successful under different circumstances.

For viruses in which there is no polymerase included in the capsid, a virion containing a minus strand is not infectious because it cannot be replicated or translated. However, a virion containing a plus strand can immediately be translated, which allows synthesis of the RdRp and subsequent RNA replication. Plus-strand viruses therefore have an obvious advantage. However, a virion containing a minus strand is still better than nothing, because it can still be replicated if it infects the same cell as a plus strand. This becomes relevant at high MOI, when most cells will be simultaneously infected by more than one virion. We have shown that a B strategy that packages both strands can outcompete a P strategy that packages only plus strands if the MOI is sufficiently high. The reason for the success of B viruses at high MOI is similar to the reason for success of bipartite viruses at high MOI in our previous work [36]. Although neither half of a bipartite virus is infectious by itself, at high MOI there is a high probability that both halves will infect the same cell.

The value of MOI that is required for the B strategy to be successful is dependent on the ratio of RNA strands to capsids produced in a cell. When RNA strands are in excess over the number of available capsids, it is advantageous not to package minus strands because this allows all the available capsids to be used for infectious plus strands. However, if the number of capsids exceeds the number of strands, then there is no benefit to not packaging minus strands, because this will not increase the number of plus-strand virions. In the latter case, it is advantageous to package both strands, because a minus-strand virion is still better than nothing. The principal result of models 1 and 2 is that if RNA strands are in significant excess over capsids, a B strategy can only win at very high MOI. The fact that plus-strand viruses are frequent in nature and B viruses are not found suggests that RNA strands are indeed in excess over capsids in real viruses, and that very high MOI values are not common in real viruses. We note that each virus capsid requires multiple proteins, and protein production is costly to the cell; therefore, it seems reasonable that the number of RNA strands that a cell can produce should exceed the number of available capsids.

Our model 3 for double-strand viruses shows that there is no benefit to producing single minus strands from the double strand, and that the only minus strands should be part of double strands. This appears to be the case for real double-strand viruses. This also highlights the similarity between double-strand viruses and plus-strand viruses that use a double-strand replicative form [3].

One of our main reasons for beginning this work was that we wanted to understand the reason for the success of minus-strand viruses. It is clear that a minus-strand virus requires a polymerase to be included in the capsid, but we have shown that this alone is not sufficient, because when both plus and minus virions are infectious, it is advantageous to package both strands. The reason that minus-strand viruses package only minus strands is that plus-strand virions are in fact not infectious when there is a distinction between replicative strands coated by a nucleoprotein and translatable strands that are not coated. A minus-strand virion is infectious because the minus strand can be transcribed to a plus strand and then translated, whereas a plus strand that is coated by the nucleoprotein cannot be transcribed and cannot be replicated without prior synthesis of the nucleoproteins. This feature of the life cycle of real minus-strand viruses unexpectedly turns out to be essential for the success of minus-strand viruses.

## Figures and Tables

**Table 1:** Reaction steps in model 1.

	Steps	Rates
1	$X^{+}\to X^{+}+X^{-}$	$kX^{+}f_{1}(R/R_{0})$
2	$X^{-}\to X^{-}+X^{+}$	$kX^{-}f_{1}(R/R_{0})$
3	$X^{+}\to X^{+}+R$	$vX^{+}$
4	$X^{+}\to X^{+}+C$	$vX^{+}$
5	$X^{+}+C\to V^{+}$	$aX^{+}\;f_{4}(C/C_{0})$ for both *B* and *P* strategies
6	$X^{-}+C\to V^{-}$	$aX^{-}\;f_{4}(C/C_{0})$ for *B* strategies, or 0 for *P* strategies

**Table 2:** Viruses produced by a single cell according to the RBP approximation.

Input State	$S_{max}$	Total Strands				Viruses Produced		
		$S_{B}^{+}$	$S_{B}^{-}$	$S_{P}^{+}$	$S_{P}^{-}$	$V_{B}^{+}$	$V_{B}^{-}$	$V_{P}^{+}$
B only	100	50	50	0	0	50	50	0
	150	75	75	0	0	50	50	0
	200	100	100	0	0	50	50	0
P only	100	0	0	50	50	0	0	50
	150	0	0	75	75	0	0	75
	200	0	0	100	100	0	0	100
B and P	100	25	25	25	25	25	25	25
	150	37.5	37.5	37.5	37.5	33.3	33.3	33.3
	200	50	50	50	50	33.3	33.3	33.3

**Table 3:** Viruses produced by a single cell in model 1. Numbers are shown for a time *t* = 100, averaged over a population of 1000 independent cells.

Input State	$S_{max}$	Total Strands				Viruses Produced		
		$S_{B}^{+}$	$S_{B}^{-}$	$S_{P}^{+}$	$S_{P}^{-}$	$V_{B}^{+}$	$V_{B}^{-}$	$V_{P}^{+}$
B only	100	49.2	48.8	0	0	47.2	46.7	0
	150	72.5	72.4	0	0	47.6	47.4	0
	200	98.1	98.0	0	0	48.6	48.5	0
P only	100	0	0	56.2	43.1	0	0	56.2
	150	0	0	85.7	62.6	0	0	85.7
	200	0	0	116.2	82.5	0	0	97.1
B and P	100	21.2	21.0	33.5	26.1	21.0	20.8	33.5
	150	30.4	30.4	51.9	38.6	23.8	23.8	48.0
	200	39.7	39.5	70.0	51.1	24.2	24.1	49.6

**Table 4:** Reaction steps in model 2.

	Steps	Rates
1	$X^{+}+R+N\to D$	$bX^{+}f_{1}(R/R_{0})f_{4}(N/N_{0}$)
2	$X^{-}+R+N\to D$	$bX^{-}f_{1}(R/R_{0})f_{4}(N/N_{0})$
3	$D\to D+X^{+}$	kD/2 for BB and BP, and kD for PP
4	$D\to D+X^{-}$	kD/2 for BB and BP, and 0 for PP
5	$X^{+}\to X^{+}+R$	$vX^{+}$
6	$X^{+}\to X^{+}+N$	$vX^{+}$
7	$X^{+}\to X^{+}+C$	$vX^{+}$
8	$X^{+}+C\to V^{+}$	$aX^{+}f_{4}(C/C_{0})$ for all strategies
9	$X^{-}+C\to V^{-}$	$aX^{-}f_{4}(C/C_{0})$ for BB, and 0 for BP and PP

**Table 5:** Reaction steps in model 3.

	Steps	Rates
1	$X^{+}+R+N\to D$	$bX^{+}{f_{1}(R/R_{0})f}_{4}(N/N_{0}$)
2	$X^{-}+R+N\to D$	$bX^{-}{f_{1}(R/R_{0})f}_{4}(N/N_{0})$
3	$D\to D+X^{+}$	kD/2 for DB and kD for DP
4	$D\to D+X^{-}$	kD/2 for DB and 0 for DP
5	$X^{+}\to X^{+}+R$	$vX^{+}$
6	$X^{+}\to X^{+}+N$	$vX^{+}$
7	$X^{+}\to X^{+}+C$	$vX^{+}$
8	$D+C\to V_{D}$	$aD\;f_{4}(C/C_{0})$

**Table 6:** Reaction steps in model 4A.

	Steps	Rates
1	$X^{+}\to X^{+}+X^{-}$	$kX^{+}f_{1}(R/R_{0})$
2	$X^{-}\to {X^{-}+X}^{+}$	$kX^{-}f_{1}(R/R_{0})$
3	$X^{+}\to X^{+}+R$	$vX^{+}$
4	$X^{+}\to X^{+}+C$	$vX^{+}$
5	$X^{+}+R+C\to V^{+}$	$aX^{+}\;f_{1}(R/R_{0})f_{4}(C/C_{0})$ for *B* and *P* strategies, and 0 for *M*
6	$X^{-}+R+C\to V^{-}$	$aX^{-}\;f_{1}(R/R_{0})f_{4}(C/C_{0})$ for *B* and *M* strategies, and 0 for *P*

**Table 7:** Reaction steps in model 4B.

	Steps	Rates
1	$Y^{-}\to Y^{-}+X^{+}$	$kY^{-}$
2	$Y^{-}+R+N\to {Y^{-}+Y}^{+}$	$kY^{-}{f_{1}(R/R_{0})f}_{4}(N/N_{0})$
3	$Y^{+}+R+N\to Y^{+}+Y^{-}$	$kY^{+}f_{1}(R/R_{0})f_{4}(N/N_{0})$
4	$X^{+}\to X^{+}+R$	$vX^{+}$
5	$X^{+}\to X^{+}+N$	$vX^{+}$
6	$X^{+}\to X^{+}+C$	$vX^{+}$
7	$Y^{+}+C\to V^{+}$	$aY^{+}\;f_{4}(C/C_{0})$ for *B* and *P* strategies, or 0 for *M* strategy.
8	$Y^{-}+C\to V^{-}$	$aY^{-}\;f_{4}(C/C_{0})$ for *B* and *M* strategies, or 0 for *P* strategy.

## Data Availability

No new data were created.
